# Laparoscopic-first approach for treating a small bowel injury in a patient stabilized after initial hemodynamic shock: a case report and video

**DOI:** 10.1097/RC9.0000000000000405

**Published:** 2026-03-26

**Authors:** Shoji Takagi, Yuta Nobunaga, Mikoto Shimabara, Yoshifumi Mitani, Masaaki Akai, Motohiko Yamada

**Affiliations:** Department of Gastroenterological Surgery, Japanese Red Cross Okayama Hospital, Okayama, Japan

**Keywords:** abdominal trauma, case report, emergency surgery, laparoscopy, penetrating injury, small bowel perforation

## Abstract

**Introduction:**

Laparotomy is the standard treatment for penetrating abdominal trauma; however, the role of laparoscopy is expanding. We report the case of a penetrating jejunal injury in a patient who initially presented with shock but was successfully managed using a laparoscopic-first approach.

**Case presentation:**

A 61-year-old male with a history of distal gastrectomy presented with a self-inflicted abdominal stab wound and had ingested antihypertensive drugs and cigarettes. He was initially diagnosed with hemorrhagic shock (systolic blood pressure, 60–70 mmHg), which stabilized after fluid resuscitation. Computed tomography revealed a hemoperitoneum, free air, and omental herniation. A laparoscopic-first approach identified 1200 mL of hemoperitoneum and a full-thickness jejunal perforation. The small bowel was inspected using the “running the bowel” technique, and the perforation was repaired with a two-layer handsewn technique. Therefore, the hybrid laparoscopic/anterior open approach was deemed useful. The patient recovered uneventfully.

**Discussion:**

This case suggests that a laparoscopic-first approach can be safely applied to select patients who initially present with transient hemodynamic instability but stabilize after resuscitation. Careful patient selection and systematic exploration are critical to avoid missed injuries. A pragmatic hybrid approach preserves the benefits of minimal invasion.

**Conclusion:**

The laparoscopic-first approach is a safe and feasible option for treating small-bowel injuries in selected patients who achieve hemodynamic stability after resuscitation. This strategy facilitates faster recovery and is particularly beneficial in selected patients requiring rapid postoperative recovery, such as those with psychiatric comorbidities.

## Introduction

Penetrating abdominal trauma is a life-threatening condition requiring urgent surgical intervention. For decades, mandatory exploratory laparotomy has been the gold standard for controlling hemorrhage and repairing visceral injuries[1]. However, laparotomy is associated with substantial morbidity, including surgical site infections, prolonged ileus, and incisional hernias^[^2,3^]^. In recent years, the laparoscopic-first approach has gained acceptance in hemodynamically stable patients, offering the advantages of minimally invasive surgery, such as reduced postoperative pain, shorter recovery times, and fewer complications^[^4,5^]^.

HIGHLIGHTS

- Laparoscopy is feasible in selected abdominal trauma patients after stabilization from initial shock.
- Systematic “running the bowel” is essential to prevent missed small bowel injuries.
- A pragmatic hybrid laparoscopic and targeted anterior open approach is useful for complex injuries.

We report a case of a self-inflicted abdominal stab wound with jejunal perforation that, despite presenting initially with shock, was successfully treated with a laparoscopic-first approach following resuscitation. This case report was prepared in accordance with the SCARE checklist[6].

## Case presentation

**Figure 1.**
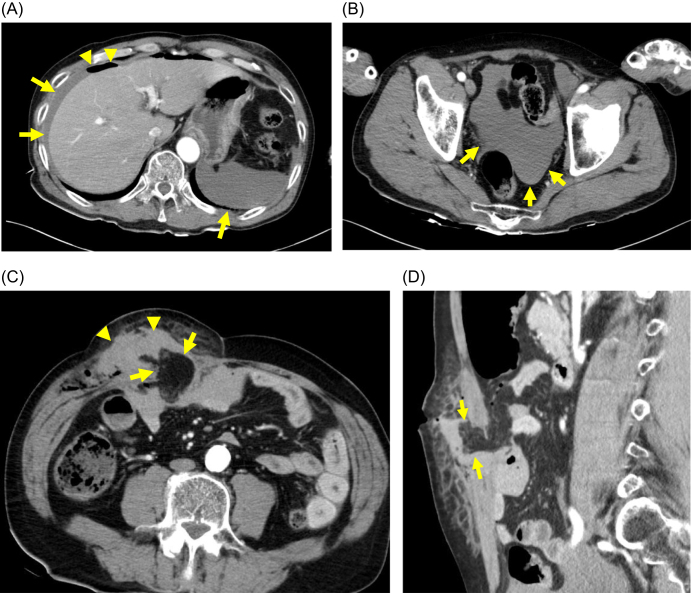
Preoperative computed tomography (CT) images. (A) Axial view showing intra-abdominal free air (arrowhead) and fluid collection (arrow). (B) Axial view showing fluid collection (arrow) in the pelvic space. (C) Axial view at the level of the stab wound showing a large abdominal wall hematoma (arrowhead) and the incarcerated omentum (arrow). (D) Sagittal view showing omental herniation through the fascial defect (arrow).

A 61-year-old male presented to our emergency department approximately 3 hours after a suicide attempt with a self-inflicted abdominal stab wound and ingestion of 6 days’ worth of antihypertensive medication and two cigarettes. He had no significant psychiatric history. He had a history of distal gastrectomy for a perforated gastric ulcer. On the scene, he was found to have hemorrhagic shock with a systolic blood pressure (BP) of 60–70 mmHg, and fluid resuscitation was immediately initiated. His hemodynamic status improved over time: 30 minutes after the start of resuscitation, the heart rate (HR) was 89 beats/min with a BP of 86/47 mmHg; at 60 minutes, the HR dropped to 81 beats/min with a BP of 96/79 mmHg. Upon arrival at our hospital, his vital signs were stable (BP, 99/64 mmHg; HR, 77 beats/min). Physical examination revealed a 10 cm transverse stab wound in the right upper abdomen and multiple injuries to the head, chest, and abdomen. Initial laboratory findings were significant, with a white blood cell count of 12 210/µL, hemoglobin of 9.7 g/dL, hematocrit of 27.9%, platelet count of 183 000/µL, and a lactate level of 45 mg/dL. A total of approximately 1500 mL of crystalloid (including pre-hospital administration) was administered prior to the computed tomography (CT) scan. Vasopressors were not required. Contrast-enhanced CT revealed a large abdominal wall hematoma, significant hemoperitoneum with free air, and omental herniation through a fascial defect (Fig. 1).

**Figure 2.**
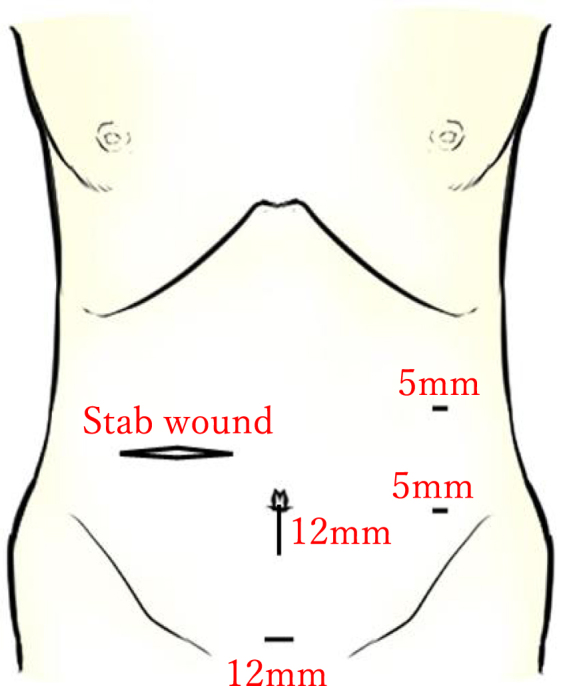
Port settings: a 12 mm trocar was inserted at the umbilicus, a 12 mm in the suprapubic region, and two 5 mm trocars in the left lateral abdominal region.

**Figure 3.**
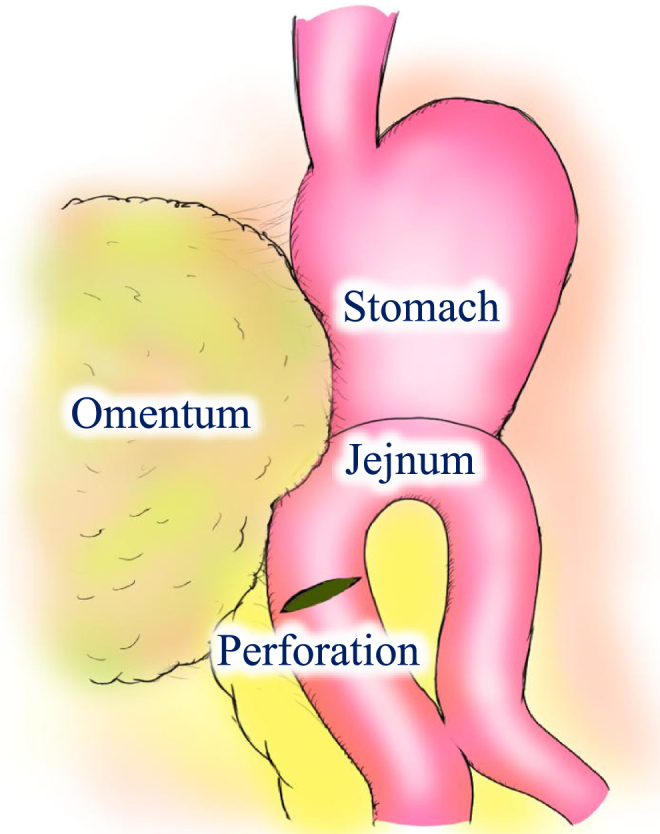
Intraoperative findings. Schematic illustration of the jejunal perforation site, located between the gastrojejunostomy and the Braun anastomosis.

Considering the patient’s hemodynamic stability after resuscitation, a laparoscopic-first approach was selected. The time from arrival to the start of surgery was 127 minutes. By the induction of anesthesia, total fluid administration had reached approximately 2000 mL. The procedure was performed by a surgical resident with 2 years of surgical specialty training and a surgeon certified under the Endoscopic Surgical Skill Qualification System (ESSQS) of the Japan Society for Endoscopic Surgery. Under general anesthesia, a 12-mm infraumbilical port was placed, with three additional working ports (two 5-mm ports and one 12-mm port; Fig. 2). A camera was inserted through the suprapubic port to provide an overview of the abdominal cavity. Laparoscopic exploration revealed approximately 1200 mL of aspirated hemoperitoneum. The entire small intestine was meticulously inspected from the terminal ileum toward the gastrojejunostomy site using the “running the bowel” technique. A full-thickness jejunal perforation was identified between the gastrojejunostomy site and Braun anastomosis (Fig. 3). The perforation was closed with a two-layer laparoscopic handsewn 4-0 absorbable suture, using a running suture for the full-thickness layer and interrupted sutures for the seromuscular layer. Intraoperatively, two units of packed red blood cells were transfused.

After reducing the incarcerated omentum, a large fascial defect compromised the pneumoperitoneum. Consequently, an anterior open approach via the stab wound was used to control active bleeding from the inferior epigastric vessels by suture ligation and repair of the transected rectus abdominis muscle. The abdomen was reinsufflated to confirm hemostasis, and a drain was placed in the Douglas pouch under laparoscopic guidance (Supplemental Digital Content Video, available at: http://links.lww.com/IJSCR/A26).

The postoperative course was uneventful. The patient began fluid intake on postoperative day 1 and diet on postoperative day 2. He advanced to a soft diet and was transferred to a psychiatric facility on postoperative day 5.

## Discussion

Previous systematic reviews have demonstrated the safety of laparoscopy for penetrating abdominal trauma in hemodynamically stable patients^[^1,2^]^. However, its application in patients with initial hemodynamic instability remains debated, although isolated cases have been reported[7]. According to the 2023 World Society of Emergency Surgery Cesena guidelines, laparoscopy is endorsed for patients with hemodynamically stable trauma, a category that includes those who are stabilized following initial resuscitation[4]. This case offers additional clinical support for the concept that the indications for this minimally invasive strategy may be cautiously extended to this specific subgroup of patients (“responders to resuscitation”), provided that rigorous patient selection and systematic exploration are maintained.

The decision to prioritize a laparoscopic approach was based on careful risk-benefit assessment. Despite the initial hypotension, our patient responded to resuscitation and achieved hemodynamic stability with an HR consistently below 100 beats/min and systolic BP maintained around 90–100 mmHg without vasopressors. Furthermore, contrast-enhanced CT revealed no active extravasation. Although a significant abdominal wall hematoma with omental herniation was present, it appeared to be tamponaded, and the absence of focal hematomas around the mesentery suggested that major vascular injury was unlikely. These findings justified the diagnostic laparoscopy, outweighing the risks of a slight delay in definitive control.

Laparoscopy has been shown to shorten hospital stays and reduce surgical site infections and pulmonary complications compared with laparotomy^[^1,2,4^]^. However, a major concern is the potential for missed injuries, underscoring the importance of a systematic exploration protocol^[^5,8^]^. As described by Kawahara *et al*[9], “running the bowel” is essential to ensure complete evaluation of the small intestine. In our case, the patient’s history of distal gastrectomy and Braun anastomosis posed a challenge. While standard “running the bowel” typically proceeds from the ligament of Treitz to the terminal ileum, we adopted a retrograde approach due to the altered anatomy. Technically, the insertion of an oblique-viewing laparoscope through the suprapubic port was a key factor that facilitated this procedure. We inspected the entire small intestine starting from the terminal ileum and proceeding proximally toward the gastrojejunostomy intracorporeally, ensuring that no injuries were missed.

Our case also illustrates the value of a pragmatic hybrid strategy. Generally, a hybrid approach involves identifying the injury laparoscopically and exteriorizing the bowel through a small incision for extracorporeal repair. However, in our case, the injured bowel could not be exteriorized due to adhesions preventing mobilization. Consequently, we performed a complete intracorporeal laparoscopic repair of the perforation. Finally, we utilized a targeted anterior open approach to repair the abdominal wall injury. This method allowed for secure control of the inferior epigastric vessel bleeding and repair of the complex rectus abdominis muscle transection, which would have been technically difficult to manage laparoscopically, while avoiding the morbidity of a full laparotomy.

In addition, adequate surgical expertise in advanced laparoscopic techniques is a fundamental prerequisite for safe application of minimally invasive approaches. When performed by an experienced surgeon, laparoscopy is a safe and feasible option even in complex trauma settings. In Japan, procedures performed by surgeons certified under the ESSQS of the Japan Society for Endoscopic Surgery are generally considered to ensure high technical standards and patient safety^[^10,11^]^.

There are several limitations to this report. First, the follow-up period was limited to the early postoperative phase due to the patient’s transfer to a psychiatric facility. Second, the successful management of this case relied heavily on the operator’s expertise; therefore, the safety and feasibility of this approach may not be generalizable to institutions without equivalent advanced laparoscopic capabilities. Finally, as a single case report, these findings require validation through larger cohort studies.

## Conclusion

The laparoscopic-first approach is a safe and feasible option for treating small-bowel injuries in selected patients who achieve hemodynamic stability after resuscitation. This strategy facilitates faster recovery and may be particularly beneficial in selected patients requiring rapid postoperative recovery, such as those with psychiatric comorbidities.

## Data Availability

The data that support the findings of this study are available from the corresponding author upon reasonable request.

## Supporting video

The surgical video demonstrates the key steps of the procedure, including laparoscopic exploration, “running the bowel” technique, and identification and repair of jejunal perforation.
